# Smoking and Looked-After Children: A Mixed-Methods Study of Policy, Practice, and Perceptions Relating to Tobacco Use in Residential Units

**DOI:** 10.3390/ijerph13060593

**Published:** 2016-06-15

**Authors:** Lisa Huddlestone, Catherine Pritchard, Elena Ratschen

**Affiliations:** 1 UK Centre for Tobacco and Alcohol Studies, Division of Epidemiology & Public Health, University of Nottingham, Nottingham NG5 1PB, UK; catherine.pritchard@nottingham.ac.uk; 2 Department of Health Sciences, University of York, York YO10 5DD, UK; elena.ratschen@york.ac.uk

**Keywords:** smoking, smoking cessation, looked-after children, residential care

## Abstract

Despite the implementation of smoke-free policies by local authorities and a statutory requirement to promote the health and well-being of looked-after children and young people in England, rates of tobacco use by this population are substantially higher than in the general youth population. A mixed-methods study, comprising a survey of residential care officers in 15 local authority-operated residential units and semi-structured, face-to-face interviews with residential carers in three local authority-operated residential units, was conducted in the East Midlands. Survey data were descriptively analysed; and interview data were transcribed and analysed using thematic framework analysis. Forty-two care officers (18% response rate) completed the survey, and 14 participated in the interviews. Despite reporting substantial awareness of smoke-free policies, a lack of adherence and enforcement became apparent, and levels of reported training in relation to smoking and smoking cessation were low (21%). Potential problems relating to wider tobacco-related harms, such as exploitative relationships; a reliance on tacit knowledge; and pessimistic attitudes towards LAC quitting smoking, were indicated. The findings highlight the need for the development of comprehensive strategies to promote adherence to and enforcement of local smoke-free policy within residential units for looked-after children and young people, and to ensure appropriate support pathways are in place for this population.

## 1. Introduction

Although comprehensive smoke-free legislation and public health guidance have reduced rates of smoking among youth in the United Kingdom (UK) over recent decades [1,2,3], tobacco smoking among looked-after children and young people, referred to as young people from hereon in, remains high. Smoking prevalence reaches up to 69% for looked-after young people accommodated in residential units, in comparison to 3% of young people aged 11–15 years within the general population [3,4]. Research suggests that, while the factors that contribute to initiation and maintenance of smoking among looked-after young people may not differ significantly from those experienced by youth in the general population, the impact of smoking may be particularly significant due to the increased vulnerability of young people looked-after [5]. In England, the term ‘looked after children’ is defined in law under the Children’s Act 1989. It refers to a child (under 10 years) or a young person (aged 10–18 years), that are provided, by way of a court order with care and/or accommodation by a local authority. This accommodation may comprise living with birth parents, with foster carers, or in a residential unit or group home. Generally, children over the age of ten years are placed within residential units when fostering provision fails or if the young person’s needs are best met within a residential setting [6]. Children and young people typically enter care due to complex family circumstances, from socially disadvantaged backgrounds, and often experience mental health problems, and which can result in significant disruptions of educational and social networks [6]. As a result of these often disparate circumstances, population-based health promotion often fails to reach this vulnerable group, and contributes to the poor health and social outcomes, such as early parenthood, unemployment, involvement with the criminal justice system, or homelessness, experienced by young people who leave care and move to independent living [5,6].

From 1 July 2007, smoke-free legislation in England prohibited smoking in enclosed or substantially enclosed work and public spaces, including vehicles. Under this law, local authorities are required to ensure that all premises associated with the local authority, including residential units for looked-after young people, are smoke-free. As the “corporate” parent, a local authority has a legal duty to ensure that young people live in a healthy environment and that their health needs are identified and services are provided to meet them, according to statutory guidance for promoting the health and well-being of looked-after children and young people [7]. While this guidance does not specifically mention tobacco smoking, public health guidance issued by the UK National Institute for Health and Clinical Excellence (NICE) sets the identification of smokers and treatment of tobacco dependence as a gold standard for various agencies and organisations, including local authorities [8]. However, a number of studies have reported that exemptions are frequently granted [6,9]. For example, a survey conducted in Scotland reported a culture within residential units for looked-after children and young people that facilitated tobacco use [9] and, more recently, observations conducted in England found smoking by young people in the grounds of residential units to be common place, with little evidence of anti-smoking messages, and staff concerns largely focusing on other self-harming behaviours [6]. Statutory health assessments are provided to young people in order to regularly review their physical, emotional and health needs, to identify any appropriate action, and to secure medical, dental and other health services needed to meet their needs [7,10]. However, despite this opportunity, tobacco use by looked-after young people often remains undetected or unaddressed by the health assessment, particularly in the presence of concerns regarding illicit substances, developmental needs, or as a result of a lack of engagement by young people in taking up offers of treatment for tobacco dependence [8,11].

In the context of disproportionate rates of tobacco use among residentially accommodated young people, tobacco-related inequality, an absence of guidance specifically targeting tobacco use by the looked-after population, and a scarcity of previous research, it is important to improve understanding of tobacco use and its management within residential units for looked-after young people. We present findings from a mixed-methods study that aimed to examine tobacco use, its impacts, current practice, and barriers and facilitators to discouraging smoking initiation and addressing the use of tobacco within residential units for looked-after young people from the perspective of residential carers.

## 2. Materials and Methods

### 2.1. Setting and Participants

The participating local authorities were located in the East Midlands region of England. Two-hundred and thirty-seven residential care officers (RCOs) working in 14 residential units for looked-after children and young people, across four local authorities, were invited to take part in the survey. Qualitative interviews were conducted in three residential units in a large rural county that provides accommodation for up to 18 young people.

### 2.2. Recruitment and Procedures

Study procedures and materials were granted ethical approval by the University of Nottingham Faculty of Medicine and Health Sciences Research Ethics Committee (REC) (references: A11092014, J15012015, and A14052015). Research governance permissions were provided by the Directors of Children’s Services in each local authority. Local authorities in the East Midlands region of England (*n* = 8) were identified using data contained in the Department for Education Children’s Homes Data Pack 2013 [11]. Prior to approaching each authority, to invite participation in the survey, approval was sought and obtained from the Association of Directors of Children’s Services (ADCS) Research Group (reference: RGE140922). From the authorities contacted, four provided permission to conduct the survey, two declined approval due to issues of capacity, and two did not respond to the application. Two-hundred and thirty-seven residential care officers (RCOs) working in 14 residential units for looked-after children and young people, across four local authorities were eligible to take part in the survey. Surveys were distributed using a variety of methods, depending on the preference of the local authority, and included distribution in person by the researcher or by the unit manager with an accompanying pre-paid envelope for survey return (*n* = 108), or via email by service managers (*n* = 129). Questionnaires were accompanied by a standard letter explaining the purpose of the study. All respondents provided informed consent, implied by completion and return of the questionnaire. The cross-sectional survey was conducted between October and December 2014.

For the semi-structured interviews, a stratified sampling strategy aimed for the inclusion of participants with a variety of ages and length of time in role, a representation of both genders, and a size of sample that would allow for a range of participant views and perceptions [12]. Managers and RCOs in three local authority operated residential units were contacted by the researcher to inform them of the study and invite participation. Participants provided written informed consent and structured data relating to participant characteristics were collected. Interviews were conducted between April and October 2015 and lasted 25–65 min, took place in a private room, and were digitally recorded. Accounts were generated from participants, using conversational prompts to allow for the emergence of novel themes. Summation was employed during discussions to ensure researcher understanding and to provide participants with the opportunity to correct any accounts, thus improving the accuracy and validity of accounts [13].

### 2.3. Instruments and Measures

For the survey, a 36-item questionnaire was developed, based on a search of the relevant literature and a survey previously conducted within a Scottish service for looked-after and accommodated children and young people [6,9,14]. It was designed to examine individual smoking behaviours, including nicotine dependence as assessed by the Heaviness of Smoking Index (HSI) [15], tobacco use during working hours, awareness of and adherence to smoke-free policies, the impact of tobacco on the day-to-day operation of the unit, smoking-related attitudes, and professional practice to address tobacco use. While it was not possible to pilot the survey, given the relatively small sample, consultation with children’s social care took place to ensure the appropriateness of the terms used. Adopting a sequential design and seeking to explore the findings from the survey, in greater depth, semi-structured interviews were conducted with residential unit managers and residential carers. Interviews were guided by a schedule of topics, developed from the findings from the cross-sectional survey conducted by the researchers, and a review of the relevant literature. Topics for discussion included: policies relating to smoking within the unit; experiences of tobacco use by young people and the delivery of advice and support for smoking cessation; tobacco-related knowledge and training; and practice and strategies to address tobacco use, including barriers and facilitators. 

### 2.4. Data Analysis

Statistical analysis of survey data was performed using IBM SPSS for Windows (Version 22) (IBM Corp, Armonk, United States). We employed descriptive statistics (frequency, proportions, median with inter-quartile range (IQR)), and Pearson’s chi-squared tests to determine if participation in training and smoking status impacted respondents’ tobacco-related attitudes or practice. Pearson’s chi-squared tests (Fisher’s exact tests where appropriate) were also used to test for proportional differences in the frequency of on-site smoking by staff, visitors, and young people, and the presence of adverse behaviours caused by tobacco, between units where smoking was accepted and those units where a smoke-free policy was enforced. Statistical significance was taken to be *p* ≤ 0.05. Levels of nicotine dependence were calculated for current smokers using the Heaviness of Smoking Index [15] and categorised as: 0 points = no dependence; 1 point = low dependence; 2–3 points = moderate dependence; and 4–6 points = high dependence. Structured interview data were entered and descriptively summarised in Microsoft Excel^®^. Narrative data were transcribed and themes and issues identified according to the framework approach described by Pope and colleagues [16]. Management of narrative data utilised Microsoft Excel^®^. Attention was paid to cases that deviated from common beliefs within the data as a method of increasing the integrity of the findings. The second and third researchers (E.R. and C.P., co-authors) independently coded 20% of the interview transcripts to establish coherence of the coding framework. Where disagreements in relation coding arose, these were discussed and settled through discussion. Coding consistency was high.

## 3. Results

### 3.1. Survey Results

#### 3.1.1. Survey Respondents and Individual Smoking Behaviors

Of the 237 RCOs who received an invitation to participate in the survey, 42 (18%) completed and returned questionnaires. The majority of participants were aged over 40 years (*n* = 29, 69%) and predominantly female (*n* = 25, 63%). Nine (21%) respondents reported having received smoking related training. Table 1 summarises respondents’ characteristics. Twelve (29%) participants reported being current smokers. Eight of those (67%) reported smoking during working hours, with tobacco use occurring within the grounds of the unit where respondents worked (*n* = 7, 88%), but out of sight of young people (*n* = 7, 70%). Table 1 further describes the individual smoking behaviours of RCOs.

#### 3.1.2. Smoke-Free Policy: Awareness, Adherence, and Impacts

Forty (93%) respondents were aware of smoke-free policies in the units where they worked. Fifteen (36%) RCOs reported that smoking by looked-after children and young people was permitted in their unit. Of the 41 (98%) RCOs who reported frequently observing tobacco use in the unit where they worked, 31 (76%) witnessed smoking by young people within the unit; nine (22%) observed staff smoking; and seven (17%) reported visitors smoking. Significant proportional differences in responses from participants from units where smoking was accepted and those where smoke-free policies were enforced were found in relation to onsite smoking by staff (47% *vs.* 8%, χ^2^ = 8.43, *p* ≤ 0.006) and visitors (36% *vs.* 7%, χ^2^ = 5.22, *p* ≤ 0.035). Differences in the enforcement of smoke-free policy were, however, not found in relation to reported on-site smoking by young people. Of the 39 RCOs who answered the question, tobacco use by looked-after young people was reported to be a source of behavioural tension within the units (*n* = 31, 79%). Significantly, respondents from units where smoking was accepted reported with greater frequency that tobacco caused tension within the unit than respondents from units where smoke-free policies were enforced (96% *vs.* 46%, χ^2^ = 13.3, *p* ≤ 0.001).

#### 3.1.3. Attitudes towards and Practice of Addressing Tobacco Use

Positive attitudes and practice towards addressing tobacco dependence among looked-after young people were reported by respondents; for instance of the 41 RCOs who responded to the question, 38 (92%) agreed that: “it was important to address tobacco dependence among young people”, while 28 (74%) of the 38 responses indicated that” looked-after young people had greater issues than tobacco dependence to deal with”. Respondents did not report the use of tobacco as a means to mediate the behaviour of young people within the unit where they worked (*n* = 33, 85%). Significant proportional differences were not found between the responses relating to practice and attitudes between smokers and non-smokers. Further details of respondents’ attitudes and practices towards addressing tobacco use are presented in Table 2.

### 3.2. Qualitative Findings

#### 3.2.1. Participant Characteristics

A total of 14 participants were recruited: all three unit managers and eleven RCOs. Participant characteristics are summarised in Table 3. The majority (*n* = 11; 79%) of participants had more than five years’ experience in their roles and two (18%) participants reported current smoking.

#### 3.2.2. Thematic Framework Analysis

Eight themes were identified from the transcribed interview data; six *a priori* themes comprised: tobacco policy rhetoric and operational reality; tobacco use in residential units for looked-after children and young people; sourcing of tobacco by young people; staff attitudes towards addressing tobacco use by looked-after young people; strategies adopted to address tobacco use in residential units for looked-after children and young people; and tobacco-related staff development. Two further themes emerged: the use of electronic cigarette and new psychotropic substances (“legal highs”).

##### ***Theme 1:*** Tobacco policy rhetoric and operational reality

All participants reported awareness of the local authority’s smoke-free policy, and in contrast to the findings from the survey, also described adherence to the policy by staff and visitors. However, for the young people themselves, and in spite of the smoke-free policy which prohibits tobacco use by looked-after children and young people, designated smoking areas were reported to be provided in the gardens of all three units. This provision was believed by Managers and RCOs to minimize risks in terms of safeguarding young people, and reportedly reduced young people’s involvement in anti-social or criminogenic behaviours.

“If young people have to go off of site to smoke, that increases the risk. If we had young people constantly at the top of the driveway, you would get are undesirable people thinking that there is a group of young people stood there and would want to see what was going on. We have to balance their safety with trying to enforce no-smoking”.

(Manager #3)

Smoking by young people within the units was reported to be governed by a number of “*local rules*”, which included limiting the number of young people using the “*smoking area*” at any one time, and the “*handing in*” of tobacco and smoking paraphernalia overnight. The majority of young people were perceived to comply with these rules.

##### ***Theme 2:*** Tobacco use in residential units for looked-after children and young people

Participants reported that very few young people initiated smoking after entry to the units, and expressed that the majority had taken up smoking while living with birth parents or foster carers.

“Most of the young people smoke before they come here, there is only one in the last year or so, since I have been here that has started while they had been in the unit. I think the reason he started was because he had come from home and wanted to be socially accepted. He just wanted to be one of the boys I think”.

(RCO #2)

Tobacco use within residential units was identified by participants as problematic, complex, and frustrating. For example, smoking was perceived to create environmental and health and safety risks, not only to the smoker, but also the other young people and staff within the unit.

“You get children smoking in their rooms, falling out, passing and bribing cigarettes; you get them flicking lighters, burning things, stubbing fags out here, there, and everywhere. It’s a health and safety nightmare”!

(RCO #6)

Other participants commented that tobacco caused tensions between young people and residential carers that were considered intimidating and at times violent.

“One young person came here from a home where I used to work so I already knew him. He was a big lad and if he didn’t have his tobacco he would smash things up. I do think maybe at one point we did have permission from his mum to actually supply him with tobacco, if it got really, really bad”.

(RCO#3)

Participants voiced frustration in relation to tobacco’s impact on the running of the residential unit.

“It [smoking] is an issue, and has been previously. Young people use it as an excuse for not doing things, that the really should be doing. For example, we’ve had young people saying “I can’t go to school, without a cigarette”. So sometimes, it’s very cleverly used by young people to avoid doing things that they really need to be doing”.

(Manager #1)

Tobacco use was suggested by participants to be the basis for many peer-to-peer relationships within the units, and often the cause of turbulence within friendships. Young people were also described as using tobacco as a tool to control or manipulate other young people within the unit, residential carers, or family members.

“Smoking is used to cause disruption or issues within the unit. Most of them are smoking because they can and it’s a big thing to hit out at the world and adults around them with. It [addiction to tobacco] is used as an excuse… for example if a young person has ran out of tobacco, they will kick off claiming that another young person has stolen it—they’re just trying it on with their behaviour to get us on side… to give them tobacco or ask another young person to share theirs—not that we ever would”!

(RCO #7)

Participants reported that parents used tobacco as a means of controlling their child’s behaviour or to prevent engagement in criminogenic activity.

“She [Mother] is intimidated by her son, and so she gets worried because he gets aggressive without tobacco; even though she doesn’t live anywhere close to him. He becomes so abusive, that she puts some money in to his bank, to please him, and that’s how it continues”.

(Manager #3)

##### ***Theme 3:*** Sourcing of tobacco by looked-after young people

Participants described how young people living in the units sourced tobacco with relative ease within their local community. A number of sources were identified, from which young people could obtain tobacco, including: parental supply during contact visits, or occasionally through the post to the unit; socially though sharing or selling/purchasing tobacco between peers; proxy purchases by members of the general public; or direct purchasing from retailers willing to make under-age sales or from “fag houses” in the local community. Both managers and RCOs considered young people astute in their ability to identify sources of illicit tobacco within the community in relatively short periods of time after entering the units.

“The places that they [young people] know in town where they can go and get the under the counter stuff—they’ll know all about that... Some will just go outside on the road here begging cigarettes, or they’ll go in to town, outside of the pub—it’s very dangerous, but if they are addicted…”

(RCO #1)

Young people were reported to adopt a number of risky practices when they were without the means to purchase tobacco. These included: approaching members of the public, to whom they are not known, in a variety of locations, including public houses, to “*cadge*” cigarettes; smoking previously discarded cigarettes, retrieved from pavements or public ashtrays; and young females were reported to engage in inappropriate relationships with older males through which they were provided with tobacco. Participants claimed a small number of young people had provided sexual favors in return for cigarettes; such a practice was considered prevalent by participants in other local authorities across the region.

“A lot of the time its “well, I gave ‘em a shag, cause I wanted a fag!” It’s that casual. The young people don’t see anything wrong with that—just another commodity…”

(RCO #3)

##### ***Theme 4:*** Staff attitudes towards addressing tobacco use by looked-after young people

Participants believed smoking to be harmful. However, for some tobacco use was perceived to have a positive effect, particularly in assisting young people to overcome issues with, self-esteem, attachment, or negative pre-care experiences.

“If they [young people] have experienced any type of abuse in their family home, smoking is used as a coping mechanism, that really needs to be addressed, or if they have low self-confidence, or a lack of identity we need to address those issues, perhaps before we then move on to think about giving up smoking. Those needs are greater and smoking fills the void until those problems are resolved”.

(RCO #4)

Participants reported a perceived responsibility to encourage young people to address their tobacco use. While engagement by young people in discussions regarding smoking was reported, participants questioned the impact of their practice to discourage tobacco use among young people. Reasons for this related to beliefs that: anti-smoking messages lost meaning since they were repeated “*ad nauseam*”; making offers of treatment to smokers who were not sufficiently motivated to quit added to the stigma already experienced by young people; and that young people’s engagement with cessation services was a tokenistic gesture made as a means of placating and maintaining good relations with carers. Participants expressed pessimism in relation to young people successfully quitting smoking.

“I don’t think its (smoking) something that they are afraid of or uncomfortable discussing! They’ll agree with us, probably to stay on side, but when it comes down to it (attending the smoking cessation service) they always find reasons not to go—there is always something else they need to do. These young people find it very difficult to talk to people they don’t know”!

(RCO#6)

##### ***Theme 5:*** Strategies adopted to address tobacco use in residential units for looked-after children and young people

Participants reported confidence in their ability to advise and support young people in addressing smoking. A number of strategies were reportedly adopted by participants to reduce the use of tobacco within residential units. These included positive role modelling and highlighting to young people the negative aesthetic and monetary aspects of smoking. In one unit, temporary abstinence was reported to be incentivized, through the use of adventure type short-breaks for young people, during which smoking was not permitted. Participants considered confronting young people with the adverse health impacts of tobacco use to be ineffective.

“Because of the situations they’re [young people] in with their lives, not living with their families, being moved from place to place; I really don’t think that they give a monkeys’ about their future! Everything is day to day, talking to them about smoking causing cancer, maybe in 50 years’ time—I don’t think that they can see that far ahead”!

(RCO #5)

Members of residential staff who reported current smoking, claimed to take care not to reveal their own smoking behaviour to young people within the units. Discovery of staff smoking by young people was reported to have led to incidents of harassment of staff by young people. While some participants believed that the awareness of young people of staff smoking was positive, and promoted frank discussions, the majority considered it to counter positive image that residential carers were required to portray.

“…and it can be difficult for a smoker to say to a young person “you shouldn’t be doing this” “this is bad for your health”—I think that some staff that feel uncomfortable with that double standard”.

(Manager #1)

Participants provided accounts of interactions with social care professionals and health services in relation to smoking and suggested that interest and engagement with reducing smoking was varied. A need to improve the process of sharing information about young people’s smoking status was highlighted by participants. In particular, the importance of information relating to the young person’s dependence on tobacco was identified. This was felt to be particularly important during the “*settling in phase*” due to the young person encountering limited sources of tobacco and the potential psychological challenges related to a placement change, that may be confounded by nicotine withdrawal.

“Some social workers will tell you ”oh they don’t smoke that much!” and suddenly you find that most of their [young person]challenging behaviours are around the fact that haven’t got a cigarette—so they must smoke a fair amount, because their behaviour tells you they’re desperate”.

(Manager #1)

The statutory health assessment for looked-after children and young people provides an opportunity for addressing tobacco dependence. However, given the additional needs of residentially accommodated young people, participants described smoking as being overlooked, particularly in the context of wider health needs.

“During the health assessments, they’ll [specialist nurses for looked-after children and young people] ask “do you smoke?”, and if yes they just put a ‘Y’ in the box and then it’s on to the next bit...”

(Manager #2)

Links with local health services including smoking cessation teams were reported to be positive, with joint working and an understanding of the complexities of “*being in care*” being displayed by local smoking cessation advisors. Such attributes were suggested by participants to allow young people to engage with services, if they wish and on their own terms. A proactive approach to limiting young people’s access to tobacco was also adopted. Partnerships forged within the local community were believed by participants to be invaluable in addressing issues of underage, proxy, or illicit tobacco purchasing.

“We work really closely with the PCSOs [Police Community Support Officers]. We build relationships between them and the young people, so that they [PCSOs] can monitor our young people—if they see them in town. With it being a fairly small town, we get a lot of telephone calls from retailers as well saying that someone [from our unit] is trying to buy cigarettes… Friends of people who work here will ring in to say one of the young people is stopping passers-by in the street [to obtain cigarettes]. That’s a big thing, if you have a community reporting back to you, then it’s a fantastic network”!

(RCO #7)

##### ***Theme 6:*** Tobacco related staff development

Despite the provision of a mandatory e-learning package in relation to smoking being available to staff, and few participants reported its completion. Among those participants who took part in the training, many felt it could be improved. In particular, participants mentioned the need to challenge staff attitudes towards smoking, the inclusion of strategies to better engage young people who believe tobacco use to be normative, and the provision of an overview of the current evidence base relating to smoking and cessation, which should include harm reduction strategies, such as electronic cigarettes and temporary abstinence.

“I think training is always useful. Compared to the e-learning, which is just a click of a mouse and yep, I’ve got my certificate; with things like health issues face to face (training) is always better as it brings out debate. You will get debate between the staff who smoke and the non-smokers”.

(Manager #1)

Participant’s knowledge of tobacco use and smoking cessation was claimed to be based upon personal experience, a tacit awareness obtained through health promotion campaigns in the media, and for many by utilizing web-based resources.

“Not training as such. But I smoke, so I know about smoking and how to give up and the things that might be helpful if I wanted to stop smoking”.

(RCO #1)

The use of informal sources of information was believed by many participants to negatively impact their ability to effectively deliver smoking related advice and support to young people accommodated within the units where participants worked.

“It’s always difficult because there’s lots of stuff [new research and practice guidance] going on—with that way is best, this way is best. It would useful for staff to be able to provide information and evidence so they are able to engage young people and to provide factual information”.

(Manager #1)

In addition to basic tobacco-related knowledge, undertaking in-depth training in smoking cessation was considered to be of benefit by participants, particularly when working with young people who were perceived as difficult to engage.

“Often the children, especial the ones we look after can often be quite intimidated by going in to a formal setting like the doctors or something like that. If we had someone in the home, who was trained, who was a constant, and who could give guidance to other staff—it wouldn’t necessarily mean that they would have to do everything, but they could give guidance to key workers”!

(Manager #3)

##### ***Theme 7:*** Electronic cigarette use

Electronic cigarette use was discussed by participants across all three units. Concerns were expressed in relation to the safety of products, including the potential health implications and the media reported risk of fire. Other concerns related to the tensions that electronic cigarette use reportedly caused within the units.

“One of our smokers has one of those bloody vape things! I don’t agree with them at all, I think they are disgusting… there is not enough research into them. I think they are just as bad as cigarettes, if not more so. People use them more and they are flavored for god’s sake! They’re like Alco-pops used to be and they should be banned”!

(RCO #3)

##### ***Theme 8:*** New Psychotropic Substances (legal highs)

Defined by the UK Home Office’s expert panel review in October 2014, new psychotropic substances (NPS) are “psychoactive drugs, newly available in the UK, which are not prohibited by the United Nations Drug Conventions, but which may pose a public health threat comparable to those substances listed in those conventions. The key features are that NPS are psychoactive, in that they stimulate or suppress the nervous system, or cause dependence; have a comparable level of potential harm to internationally controlled drugs; and are newly available, rather than newly invented”. Legal highs were mentioned by participants from two units. Frustration was expressed by staff in being unable to remove the substances from LAC, given that possession of such substances is not illegal. Others highlighted the contribution of legal highs to tobacco-related adverse behaviours within the units and the impact on the capacity of the unit as a whole.

“(Young person) doesn’t only smoke tobacco, but legal highs also. It creates a massive issue for us and we do get the flips between being completely unconscious and his additional needs in terms of being monitored to really violent outbursts because he is coming down and really needing tobacco and that’s when he becomes violent—harassing people, and banging on doors to get people up because he knows that they have tobacco”.

(Manager #2)

## 4. Discussion

This study demonstrates that tobacco smoking is highly prevalent in residential units for looked-after children and young people, and has wide ranging impacts that include exposing young people to the risks of exploitation, psychological harm, and physical health risks that exceed those of tobacco smoking, and appear to be rarely addressed in appropriate ways. Our findings convey the necessity to develop an intervention that takes account of the factors that challenge or facilitate the reduction of tobacco use within residential units for looked-after children and young people.

### 4.1. Adherence to and Effect of Smoke-Free Policy

The findings from this study illustrate that current smoke-free policies pertaining to residential units are insufficient in discouraging or addressing tobacco use by young people. While participants expressed a substantial awareness of local authority policies that prohibit smoking on the residential premises, smoking by young people is common. With past research finding smoking among youth in the general population to be influenced by growing-up in environments in which smoking is acceptable or commonplace, this lack of adherence and enforcement of the policy and proactive health promotion is concerning [17,18,19,20,21]. Although a number of potential reasons for permitting smoking within the grounds of residential units were suggested by participants and included the prevention of young people’s involvement in crime or anti-social behaviour [22], or minimizing risks related to child sexual exploitation(CSE) [23], like any responsible parent, residential carers seek to manage the risk that young people may face, and often this requires a balance to be struck between safeguarding and an acceptance of risky health behaviour. Nevertheless, permitting smoking by looked-after youth has a number of negative implications. Given the difficulty some young people experience in developing peer relationships, smoking may facilitate this and potentially lead to the initiation of smoking, as a means of engaging with peers [6]. In addition to establishing relationships, smoking contributes to peer to peer bullying of young people, harassment of staff and other young people, and aggression within the units. Permitting smoking may also sabotage the efforts of young people contemplating or attempting cessation [24]. Finally, the finding that some residential carers smoke within the grounds of the units, may mean that, inadvertently, this behaviour is witnessed by young people, thereby adding to the normalization of tobacco use and undermining efforts to promote healthy behaviours.

### 4.2. Tobacco Use within Residential Units

Tobacco smoking by residentially looked-after young people is highlighted as complex and bound in the negotiation of power, relationships, and social belonging in our study. While these interactions are reported to some extent among youth within the general population [25], the role of tobacco in exerting power or control, social contexts and interaction is compounded among looked-after young people, given the uncertainty experienced and the difficulties encountered with building and maintaining relationships [26]. Proxy purchasing and the use of community based illicit sources of tobacco appeared to be frequently used methods by which young people acquired tobacco. Supported by previous research, these findings indicate that tobacco control policy bans on under-age purchasing are easily circumvented [27,28]. While the purchasing of tobacco from illicit sources, has not been explored in under-age smokers, findings from research with adult smokers suggest a relationship between illicit tobacco use and significantly worse health [29]. Furthermore, in addition to the risks of smoking illicit tobacco, the risk from potential exploitation or coercion is high, given the links between the black market and crime. With the advent of the Children and Families Act 2014, that came into force on 1 October 2015 in England, and which provides enforcement and fines to regulate proxy purchasing and sales of nicotine containing products to children and young people under the age of 18, the development of pathways to share intelligence with trading standards may be beneficial in reducing the supply of tobacco to looked-after young people. Family members have been identified in previous research to be a likely source of tobacco for youth [30]. With our findings illustrating the frequent occurrence of parents supplying young people with tobacco, and of looked-after young people sharing, trading and selling tobacco to peers, parents may well be indirectly supplying tobacco to other vulnerable young people. Effective engagement is therefore critical if parents are to be dissuaded from supplying their young people with tobacco or the means by which to purchase it.

### 4.3. Wider Tobacco Related Risks

Our findings highlight the desperate measures looked-after young people will resort to in order to obtain tobacco. Previous research has highlighted the practice of youth smoking cigarettes that have been discarded in the street or approaching unknown adults in order to “cadge” a cigarette [28,30]. The smoking of discarded cigarettes is particularly concerning, given that the practice places the smoker at risk of disease transmission [31]. While smoking related practices that exceed the risks of smoking have been identified within disadvantaged adult populations, such as the homeless [31], looked-after young people within this study are reported to engage in risk behaviours that have not been reported previously. Their engagement in sexual behaviour in return for tobacco and the formation of inappropriate relationships with older males is highly concerning, especially in light of recent reports of the sexual exploitation and trafficking of looked-after children and young people within the UK, by criminal gangs [32]. Approaches that promote self-esteem and protective factors for young people identified during key working and therapeutic sessions within units or in conjunction with other health professionals may be useful to address wider harms.

### 4.4. Opportunities and Challenges to Address Tobacco Use

Attitudes towards addressing tobacco dependence among looked-after young people were generally positive, and despite a lack of training in relation to smoking, levels of confidence in providing advice and support to young smokers to address their tobacco dependence were high. However, a reliance on tacit and web acquired information raises questions over the accuracy of smoking related advice provided to young people and is likely to limit the effectiveness of strategies to promote cessation. A number of participant misattributions regarding smoking were apparent. For example, the prevailing belief that looked-after young people have greater issues to deal with than addressing their tobacco use indicates a lack of awareness of the importance of smoking cessation and the benefits which can be gained. While it is widely reported that many young people in public care experience mental health problems related to their pre-care experiences [6,33], this, however, should not be considered a barrier to smoking cessation. For instance, while, motivation to quit smoking among youth with mental disorders is not well researched, one study [34] found that as many as 42% of youth in the psychiatric population report a desire to quit and two-thirds report having made a quit attempt. Similarly, within the homeless population, many of whom are reported to have spent some of their childhood in care, smoking cessation interventions have been shown to be effective, despite higher levels of nicotine dependence and co-occurring mental disorders [5]. Pessimistic attitudes of residential carers in relation to the capacity of looked-after young people to be motivated to attempt and achieve smoking cessation is likely to result in overlooked opportunities through which young people may be engaged in constructive discussions regarding smoking cessation.

### 4.5. Limitations

Our mixed-methods approach has several limitations. The response rate of 18% to the survey may mean that results may not be generalizable to residential units in other areas of England or the UK. In respect to the survey response, while relatively low, it does compare to responses achieved by other surveys, conducted within local authority social care, which utilized electronic distribution [35,36]. Despite this, the findings are important given that this is the first survey of its kind to explore tobacco use within English residential units for looked-after children and young people, from the perspective of front-line staff, across a variety of residential settings and locations. Participants were likely to be individuals with an interest in the topic, and survey selection and reporting bias may have influenced the favorable responses to survey and interview questions pertaining to participant’s confidence in their ability to support smoking cessation attempts among looked-after young people. While we attempted to explore the smoking related training participants had undertaken, this was not verified by viewing training records, and the content of reported training provided was not ascertained.

### 4.6. Further Research

Future research should involve an exploration of strategies to overcome the perceived barriers to making residential units for looked-after children and young people smoke-free, taking into account the need to minimize safeguarding risks and behavioural implications resulting from nicotine withdrawal. In the future, researchers may wish to examine the ways in which residential carers can encourage and support young people accommodated in residential units to address their tobacco dependence, particularly during times of stress or instability, and develop strategies for providing consistent, evidence based support to enable looked-after young people to view smoking cessation as positive and beneficial. Given that looked-after young people have been identified as a population who typically encounter challenges in accessing health promotion, it may be also be valuable to consider how young people view these discussions and how they are experienced, with a particular focus on the language used in the delivery of tobacco-related advice and information, and the barriers looked-after young people perceive to smoking cessation. An exploration of parental beliefs and attitudes of smoking by young people is also warranted and may also include the perceived facilitators and opportunities for parental engagement in supporting looked-after young people in smoking cessation.

## 5. Conclusions

Tobacco use within residential units for looked-after children and young people poses significant risks on various levels and presents residential carers with significant challenges. The development of cohesive smoke-free policies that are underpinned by comprehensive staff development, buy-in from parents, the local authority, and other agencies supporting young people in residential care is crucial to support smoke-free environments for these young people. The integrated nature of this may result in the need for the formulation of a complex intervention that takes account of the multiplicity of factors that result in tobacco use within residential units for looked-after young people. Addressing these issues is an important component in tackling the health inequalities experienced by looked-after young people.

## Figures and Tables

**Table 1:** Summary of participant characteristics and respondents’ smoking behaviour.

Characteristic (Number of Respondents)		Frequency (%)
Gender (*n* = 40)	*Female*	25 (63)
	*Male*	15 (37)
Age range (*n* = 42)	*20–29 years*	3 (7)
	*30–39 years*	10 (24)
	*40–49 years*	11 (26)
	*50–59 years*	17 (41)
	*60–69 years*	1 (2)
Attendance at smoking related training (*n* = 42)	*Yes*	9 (21)
	*No*	33 (79)
Smoking status (*n* = 42)	*Never smoker*	19 (45)
	*Ex-smoker*	11 (26)
	*Current smoker*	12 (29)
**Assessment of Individual Smoking Behavior**		
Daily tobacco consumption (*n* = 12)	*1–10 cigarettes*	9 (75)
	*11–20 cigarettes*	3 (25)
Level of nicotine dependence (*n* = 12)	*None*	4 (33)
	*Low*	3 (25)
	*Moderate*	5 (42)
Smoking during working hours (*n* = 12)	*Yes*	8 (67)
	*No*	4 (33)
Location of smoking during working hours (*n* = 8)	*Off site*	1 (12)
	*In the grounds of the unit*	7 (88)
Frequency smoking occurs in sight of looked-after young people (*n* = 10)	*Occasionally*	3 (30)
	*Never*	7 (70)

**Table 2:** Respondents experience, attitudes and practice relating to tobacco use and smoking cessation among looked-after young people.

Statement (Number of Responses Provided)	Agree/True	
	Frequency	%
Smoking is accepted in the unit where I work. (*n* = 42)	15	36
I often witness staff smoking on the premises. (*n* = 41)	9	22
I often witness visitors smoking on the premises. (*n* = 41)	7	17
I often witness looked-after young people smoking on the premises. (*n* = 42)	31	74
Smoking is regularly a source of tension within the unit. (*n* = 39)	31	79
Tobacco is used to negotiate the behaviour of looked-after young people within the unit. (*n* = 39)	6	15
Tobacco contributes to bullying or anti-social behaviour with the unit. (*n* = 39)	35	90
I have experiences violence or aggression from looked-after young people in relation to smoking. (*n* = 39)	35	90
It is important to address smoking among looked-after young people (*n =* 41)	38	93
It is within the remit of my role to support young people within the unit where I work in quitting smoking *(n =* 40)	38	95
looked-after young people have greater issues than tobacco use to deal with *(n = 38)*	28	74
I have enough knowledge of tobacco dependence and its treatment *(n = 38)*	31	82
I am comfortable discussing smoking with looked-after young people *(n = 39)*	38	97
I regularly provide young people in the unit where I work with advice about smoking and the benefits of quitting *(n = 39)*	35	90
I would feel confident in supporting looked-after young people in stopping smoking *(n = 39)*	39	100

**Table 3:** Summary of participant characteristics by staff group.

Participant Characteristic		Unit Managers (*n* = 3) Frequency, (%)	RCO (*n* = 11) Frequency, (%)
Unit	*A*	1 (33)	4 (36)
	*B*	1 (33)	3 (27)
	*C*	1 (33)	4 (36)
Gender	*Female*	2 (67)	8 (73)
	*Male*	1 (33)	3 (27)
Age range	*20–29 years*		3 (27)
	*30–39 years*		2 (18)
	*40–49 years*		2 (18)
	*50–59 years*		4 (36)
Smoking status	*Current smoker*		2 (18)
	*Ex-smoker*		4 (36)
	*Never smoker*	3 (100)	5 (45)
Time in role	*Less than 1 year*		1 (9)
	*1–3 years*		2 (18)
	*5 or more years*	3 (100)	8 (73)
RCO Grade	*RCO 1*		5 (45)
	*RCO 2*		1 (9)
	*RCO 3*		4 (36)
	Assistant manager		1 (9)

## Abbreviations

The following abbreviations are used in this manuscript:

ADCS	Association of Directors of Children’s Services
CSE	Child Sexual Exploitation
HIS	Heaviness of Smoking Index
LAC	Looked-After Children and Young People
NICE	National Institute of Health and Clinical Excellence
RCO	Residential Care Officer
REC	Research Ethics Committee
UK	United Kingdom
